# Heavy Lifting at Work and Risk of Ischemic Heart Disease: Protocol for a Register-Based Prospective Cohort Study

**DOI:** 10.2196/resprot.3270

**Published:** 2014-08-20

**Authors:** Harald Hannerz, Andreas Holtermann

**Affiliations:** ^1^National Research Centre for the Working Environment, DenmarkCopenhagenDenmark

**Keywords:** occupational health, cardiovascular disease, manual work, cohort study

## Abstract

**Background:**

There are theoretical grounds to suspect that heavy lifting at work is an important risk factor for ischemic heart disease (IHD). However the relationship has not been sufficiently acknowledged by empirical studies. Positive and statistically significant associations have been found in studies that utilize self-reported exposure data. Such studies are, however, prone to reporting bias. All else equal, people with a poor cardiovascular fitness/health may have a higher propensity to perceive their work environment as heavy.

**Objective:**

The study described in the present protocol aims to investigate the relationship between heavy lifting at work and IHD by use of material and methods that are free from reporting bias.

**Methods:**

This is a register-based prospective cohort study. Male blue-collar workers in Denmark will be identified and followed through national registers, from 2001-2010, for hospital treatment or death due to IHD. Relative rates of IHD between “workers in occupations likely to involve heavy lifting” and “other blue-collar workers” will be estimated through Poisson regression.

**Results:**

Results are expected to be ready in mid-2015.

**Conclusions:**

Since this is not a randomized study, it cannot confirm etiological hypotheses. It may, however, confirm that employment in occupations that involve heavy lifting is a predictor for IHD and thereby lend support to the hypothesis of a causal relationship.

## Introduction

### Background

A positive association between occupational physical demands, cardiovascular disease, and mortality has been found in several cohorts [1-8]. However, the specific types of occupational physical demands conferring the increased risk for cardiovascular disease remain unsettled [8].

A retrospective case-control study has indicated that occupational heavy lifting increases the risk for acute myocardial infarction while occupational walking and leisure time physical activity decrease the risk [3]. Moreover, in a recent prospective multi-adjusted study [9], men reporting heavy lifting at work had 55% increased risk for ischemic heart disease (IHD). Occupational heavy lifting has also been shown to impose an acute cardiovascular strain, excessively raising blood pressure for a prolonged period of time [2].

However, the two previous studies on the relationship between occupational heavy lifting and IHD may be biased by the self-reported measure of occupational heavy lifting. For example, it has been shown that people with myocardial infarction tend to remember and report previous physical activity levels differently than comparable control persons [10]. The largest recall bias was found for estimated associations between myocardial infarction and perception of occupational workload and repetitive or heavy lifting at work.

Therefore, studies investigating the risk for IHD among blue collar occupational groups likely to be involved in occupational heavy lifting compared with blue collar occupational groups not likely to be involved in occupational heavy lifting are needed.

### Objectives

The present study aims to investigate the relationship between heavy work and risk of IHD, among men in the general working population of Denmark.

The following hypotheses will be tested: (1) the rate of hospital treatment or death due to IHD is higher among blue-collar workers in occupations likely to involve heavy lifting than it is among blue-collar workers in occupations less likely to involve heavy lifting, and (2) the rate of death due to IHD is higher among blue-collar workers in occupations likely to involve heavy lifting than it is among blue-collar workers in occupations less likely to involve heavy lifting.

The above hypotheses are operationalizations designed to shed light on the following underlying hypotheses: (1) prolonged periods of occupational heavy lifting are likely to increase the risk of IHD, and (2) prolonged periods of occupational heavy lifting are likely to increase the risk of death due to IHD, either through an increased risk of occurrence or an increased risk of death given occurrence of the disease.

The hypotheses are based on the following theoretical arguments: heavy lifting is well known to acutely increase blood pressure. Several hours work per day of lifting-induced elevated blood pressure with minor possibilities for sufficient restitution may cause endothelial micro ruptures and damage of the arteries [1], inducing absorption of lipids and other pathogenic substances in the arterial wall leading to arteriosclerosis [11]. These conditions are associated with an increased risk of IHD [12].

## Methods

### Data Source

The study will use a database obtained through a record-linkage between four national registers—the central person register [13], the hospital patient register [14], the cause of death register [15], and the employment classification module. The central person register contains information on gender, addresses, and dates of birth, death, and migrations for every person who is or has been an inhabitant of Denmark sometime between 1968 and present time. A person’s occupation and industry are, since 1975, registered annually in the employment classification module. Since 1994, the occupations are coded according to the Denmark International Standard Classification of Occupations (DISCO-88) [16], which is a national version of the International Standard Classification of Occupations (ISCO-88). The national hospital register has existed since 1977 and contains data from all public hospitals in Denmark (more than 99% of all admissions). From 1977 to 1994, the register only included inpatients but from 1995 it also covers outpatients and emergency ward visits. Since 1994, the diagnoses are coded according to the International Classification of Diseases, 10th revision (ICD-10) [17].

The study will comply with The Act on Processing of Personal Data (Act No. 429 of 31 May 2000), which implements the European Union Directive 95/46/EC on the protection of individuals. The data usage is approved by the Danish Data Protection Agency, journal number: 2001-54-0180. According to Danish law, questionnaire and register-based studies do not need approval by ethical and scientific committees, nor informed consent.

### Occupational Categories

The occupational categories to be used in the hypothesis tests are given, in terms of DISCO-88 codes, in Table 1. The occupations considered to be strongly associated with heavy lifting were selected in accordance with expert opinion by Professor Holtermann, National Research Centre for the Working Environment (NRCWE). The opinion of Holtermann was seconded by Professor Karen Søgaard, University of Southern Denmark. The selection was done in connection with the design of an earlier study, which would investigate the association between heavy lifting and retinal detachment. Data from the Danish work environment cohort survey (DWECS) in the year 2000 [18] were used to confirm that the occurrence of heavy lifting among male workers who belong to Holtermann’s group is significantly higher than it is among workers who belong to the other occupational category. Responses to the following questions were considered: (1) How much of your time at work do you carry or lift objects? (Almost all the time; Approximately 3/4 of the time; Approximately 1/2 of the time; Approximately 1/4 of the time; Rarely/very little; Never), and (2) How much does what you carry or lift typically weigh? (Less than 3 kg; 3-10 kg; 11-29 kg; 30-49 kg; 50 kg or more).

The proportion who reported that they carry or lift objects approximately one-quarter of the time or more was statistically significantly higher among males in Holtermann’s group than it was among other blue-collar workers. The same holds for the ones who reported that they carry or lift objects approximately one-quarter of the time or more and that the objects typically weigh 30 kg or more.

**Table 1:** Occupational categories according to DISCO-88.

Occupational category	DISCO-88
**Holtermann’s group (occupations that are strongly associated with heavy lifting)**	
	712. Building frame and related trades workers
	921. Agricultural, forestry and fishery laborers
	931. Construction laborers
	933. Transport and storage laborers
**Other blue-collar workers (occupations in which heavy lifting is less likely to occur)**	
	All workers with a first digit DISCO-code equal to 6 (agricultural trades workers), 7 (craft and related trades workers), 8 (plant and machine operators and assemblers), or 9 (elementary occupations). Except those that belong to Holtermann’s group.

### Statistics on Lifting, Smoking, and BMI

We know that a high body mass index (BMI) and smoking are important risk factors for IHD. Unfortunately, the data material of the present study does not contain individual-based information on these factors. We have, however, individual-based data on a random sample (ca. 1 per 400) of our study population (DWECS 2000), which we can use to evaluate the possible influence of the two risk factors. In Table 2, we give estimated percentages of occupational lifting, by occupational group. We also give statistics on BMI and smoking habits.

As seen in Table 2, the proportion of overweight workers was significantly lower among workers in Holtermann’s group than it was among other blue-collar workers. To get an idea of the extent that this factor may influence the rate ratio of IHD between the two groups, we calculated the expected rate ratio under the assumption that the groups are equal in all respects other than the BMI distribution. According to our calculations, the expected rate ratio, E[RR], for IHD (Holtermann’s group vs other blue-collar workers) equals 0.96 (95% CI 0.91-1.01) under the assumption that the groups are equal in all respects other than the BMI distribution. The standard error of the logarithm of the estimate equals 0.0274. The standard error and the confidence interval are based on Gauss propagation of error formulas. The point estimate is based on the following equation:

E[RR]=(1+p_1_(RR_1_−1) + p_2_(RR_2_−1)) / ((1+q_1_(RR_1_−1) + q_2_(RR_2_−1))

where RR_1_is the rate ratio for IHD among people in the category 25≤BMI< 30 vs BMI< 25 and RR_2_is the rate ratio between the categories BMI≥30 and BMI<25. The p_i_’s are the proportions in the various BMI categories in Holtermann’s group while the q_i_’s are the proportions among other blue-collar workers. The proportions were estimated through DWECS 2000, while the rate ratio for IHD, by BMI group, was estimated on workers registered in DWECS 1990, 1995, 2000, and 2005 through a follow-up (1991-2009) in the national registers described in the Methods section. The estimated rate ratio for IHD, by BMI category is given in Table 3.

**Table 2:** Percentage of male workers exposed to occupational lifting, smoking, and high body mass index (BMI) according to DWECS 2000, by occupational category.^a^

Exposure category	Holtermann’s group n (%)	Other blue-collar workers n (%)	OR	95% CI
Lifting ≥¼ of work hours	206 (79.2)	512 (47.7)	4.17	3.02-5.76
Lifting ≥¼ of work hours (“The objects weigh typically 30 kg or more.”)	43 (16.5)	114 (10.6)	1.67	1.14-2.44
BMI ≥25	113 (43.7)	574 (53.8)	0.66	0.50-0.87
BMI ≥30	25 (9.7)	116 (10.9)	0.88	0.56-1.38
Current smoker	118 (45.4)	481 (45.0)	1.02	0.78-1.34

^a^The total number of observations varied (due to missing values) between 259 and 260 in Holtermann’s group and between 1067 and 1073 among other blue-collar workers.

**Table 3:** Rate ratio (RR) with 95% confidence interval (CI) for hospital treatment or death due to IHD, 1991-2010, by body mass index (BMI) in a representative sample of Danish employees.^a^

BMI	Persons	Person years	Cases	RR	95% CI
<25	7078	89,514	180	1.00	-
25≤ BMI< 30	4059	41,564	186	1.41	1.14-1.74
≥30	1244	10,454	87	2.69	2.08-3.49

^a^The analysis was controlled for gender, age, and calendar time.

### Statistics on Other Occupational Exposures

In the last section, we looked at BMI and smoking. In this section, we will look at statistics on certain work environmental factors, which theoretically may influence the rate of IHD. The following occupational exposures have been associated with an increased risk for subsequent IHD: noise [19], poor decision latitude [20], high job insecurity [21], shift work [22], prolonged working hours [23], and air pollution [24].

These factors are not as influential as BMI—being obese versus normal weight increases the risk with almost 200%, while the various work environmental exposures typically are associated with an elevated risk of between 5 and 25% [19]. Nevertheless, if we find that the rate of IHD is significantly higher in “occupations that are strongly associated with heavy lifting” than in “occupations in which lifting is less likely to occur”, then we want to rule out the possibility that the result is due to an increased exposure to other work environmental risk factors.

DWECS 2000 does not provide any reliable measure of exposure to air pollution. It contains, however, questions that can be used to obtain measures of the other above mentioned risk factors. In Table 4, we give estimated percentages of exposure to noise, poor decision latitude, high job insecurity, shift work, and prolonged working hours, by occupational category.

A person was categorized as exposed to:

- loud noise, if he replied “Approximately 1/4 of the work hours” or more to the question “Are you subjected to noise that is so loud that you have to raise your voice in order to talk to others?” He was categorized as unexposed if he replied “Rarely/very little” or “Never”
- poor decision latitude, if he replied “Rarely” or “Never/Almost never” to the question “Do you have a significant influence in the decision making at your work?”
- high job insecurity, if he answered “Yes” to any of the following questions: “Are you worried about becoming unemployed?” or “Are you worried about difficulties in finding a new job with your present qualifications?”
- prolonged working hours, if he worked 41 hours or more per week
- shift or night work, if the normal placement of his work was either fixed at night or shifting (working on two shifts, three shifts, or irregularly during the day/week according to special schedule or rotation). He was categorized as unexposed if he replied “Fixed day duty”, “Fixed morning duty”, or “Fixed evening shift/evening work”.

**Table 4:** Percentage of male workers exposed to loud noise, low decision latitude, high job insecurity, prolonged working hours, and shift or night work, according to DWECS 2000, by occupational category.^a^

Exposure category	Holtermann’s group n (%)	Other blue-collar workers n (%)	OR	95% CI
Loud noise ≥¼ of work hours	116 (44.6)	463 (43.2)	1.06	0.81-1.39
Poor decision latitude	76 (29.2)	331 (30.0)	0.92	0.68-1.24
High job insecurity	62 (26.4)	311 (32.2)	0.76	0.55-1.04
Prolonged working hours	46 (17.7)	318 (29.9)	0.50	0.36-0.71
Shift or night work	22 (8.5)	216 (20.2)	0.37	0.23-0.58

^a^The total number of observations varied (due to missing values) between 235 and 260 in Holtermann’s group and between 967 and 1072 among other blue-collar workers.

### Study Design

The study population consists of all male inhabitants of Denmark, who at baseline (January 1, 2001) were 21-59 years old and belonged to any of the two occupational groups given above. These people will be followed in our national registers, from January 1, 2001 to December 31, 2010. Approximately half a million working men will be included in the study.

The following clinical endpoints will be considered: (1) hospital treatment or death with IHD as principal diagnosis/cause of death (the case definition includes these ICD-10 codes: I20 angina pectoris, I21 acute myocardial infarction, I22 subsequent myocardial infarction, I23 certain current complications following acute myocardial infarction, I24 other acute ischemic heart diseases, I25 chronic ischemic heart disease), and (2) death with IHD as principal cause of death.

The study population will be followed, first for death or hospital treatment due to IHD and then for death due to IHD. Only those who were free from IHD-related hospital visits throughout the calendar year preceding baseline will be included in the analysis.

For each of the two endpoints, each of the included individuals will be followed until any of the following events occur: he reaches the clinical endpoint of the follow-up, he emigrates, he dies, or the study period ends. Person years at risk (PYRS) will be calculated for each individual.

We will use Poisson regression to estimate rate ratios between Holtermann’s blue-collar workers and all other blue-collar workers, while adjusting for age (10-year age groups). The rate ratios will be presented with 95% confidence intervals. PROC GENMOD in SAS version 9.3 will be used to implement the analysis.

### Hypothesis Testing Criteria

A hypothesis is confirmed (regarded as statistically significant) if the lower boundary of the 95% confidence interval of its associated rate ratio exceeds one. The significance level is thereby set at .025.

If the first hypothesis is confirmed and the rate ratio of the second hypothesis exceeds one, then we will conclude that the first of the underlying hypotheses is supported by the data. If the second hypothesis is confirmed, then we will conclude that the second of the underlying hypotheses is supported by the data.

### Power Calculations

A 10-year follow-up (2001-2010), including all economically active men in Denmark aged 21-59 years in January 2001, yields ca. 420 cases of hospital treatment or death due to IHD per 100,000 person years, and 23 cases of death due to IHD per 100,000 person years. At baseline, 8.73% (112,699/1,291,665) of the workforce belonged to Holtermann’s group while 31.38% (405,385/1,291,665) belonged to the group “other blue-collar workers”. If we assume that the number of cases is proportional to the number of people in the various job groups, then the expected numbers of cases of hospital treatment or death due to IHD are approximately 4650 in Holtermann’s group and 16,000 among the other blue-collar workers. The corresponding numbers for death due to IHD are 255 and 880.

**Figure 1:**
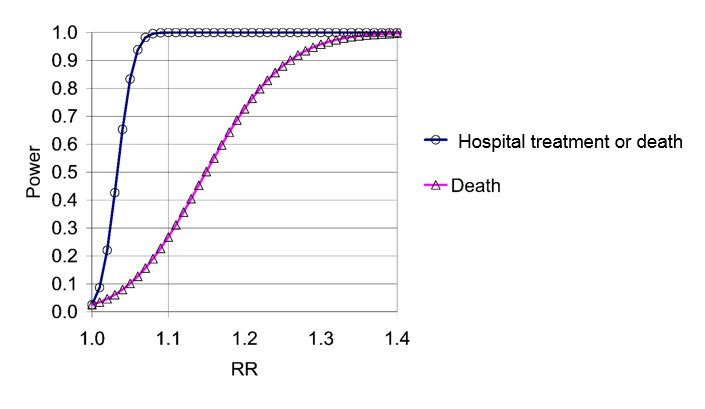
Approximate power curves for the hypothesis that the rate of IHD is higher among men in Holtermann’s group than it is among other blue-collar workers as a function of the true rate ratio (RR).

With the above data as input, the statistical powers of the tests are given as a function of the true rate ratio in Figure 1. The calculations are based on the Poisson distribution, the propagation of error formulas, and the central limit theorem.

### Sensitivity Analysis 1

Since the latency period for ischemic heart disease is long [25], a person will be kept in his baseline exposure category throughout the follow-up regardless of whether or not he shifts to another job or retires from the labor market during the follow-up.

During the study period, the participants were eligible for an old age pension at the age of 65. It was, however, also possible to retire prematurely at the age of 60, according to an optional public insurance policy. It is possible that heavy lifting at work influences the propensity to opt for early retirement. It is also possible that early retirement influences the risk of IHD. Early retirement might, in other words, be a moderating factor for the risk of subsequent IHD as a function of heavy lifting at work, among people who are 60 years or older. If this is the case, then we might not be able to generalize the results of the present study to nations or time periods with other retirement schemes.

To shed some light on this issue, we will perform a sensitivity analysis where we censor the follow-up whenever a participant reaches age 60. The clinical endpoint of the sensitivity analysis will be hospital treatment or death due to IHD as principal diagnosis/cause of death.

### Sensitivity Analysis 2

The inclusion/exclusion criteria of the primary analysis are based solely on information from the calendar year preceding baseline. It would have been possible to involve information from more than one calendar year; however, since we want our estimates to be representative of the situation among all of the concerned workers who were healthy enough to be classified as economically active at baseline, we elected our exclusion criteria parsimoniously. In the primary analysis, we exclude all workers who were treated for IHD sometime during the calendar year preceding baseline and, by doing so, we have ascertained that an observed instance of hospital treatment during the follow-up is a new episode rather than a revisit in a course of treatment that had already started before baseline. We cannot know, however, if this was the first occurrence of the disease ever.

It is possible that some workers, due to ischemic heart disease, changed from an occupation that entails heavy lifting to an occupation that does not entail heavy lifting, a few years prior to baseline. If this is the case, then, from an etiological viewpoint, our estimates will be biased. We will address this issue with a sensitivity analysis that only includes those who lived in Denmark, were free from IHD-related hospital contacts, and belonged to the same occupational category throughout a 3-year period prior to baseline. The clinical endpoint will be hospital treatment or death due to IHD. Since our project does not have access to information about people who are younger than 20 years and the inclusion/exclusion criteria are based on a 3-year period, we can only include people who were at least 23 years old at baseline. In all other respects, we will use the design of the primary analysis.

## Discussion

### Study Protocol

With this study protocol, we define the hypotheses, inclusion criteria, statistical models, and test criteria completely before we look at any relationship between the exposure and outcome variables in our data. By doing so, we have eliminated hindsight bias. The prospective design and the exclusion of prevalent cases ascertain that the exposure takes place before the outcome. The size of the study gives us a tremendous statistical precision and, since we are dealing with registers that cover the entire population of Denmark, we do not have any problems with sampling bias, response bias, or volunteer bias.

### Challenges

The drawback of the study is that it lacks individual-based data on some quite important predictors for IHD, such as smoking [26], BMI [27], physical fitness [28], blood pressure, and cholesterol [29]. It is well documented that the distribution of risk factors for IHD differs between social groups [30,31] and that the rates of IHD generally are lower among white-collar workers compared with blue-collar workers. To mitigate the possible influence from uncontrolled risk factors, we decided to only include blue-collar workers in our comparison group. The drawback of this decision is that the comparison group also, to some degree, will contain elements of heavy lifting.

We used data from a survey on a random sample of the study population to look at differences, between the group associated with heavy lifting and other blue-collar workers, in the prevalence of smoking, overweight, obesity, exposure to noise, poor decision latitude, high job insecurity, prolonged working hours, and shift work. We did not find any difference in smoking prevalence but we found that the prevalences of overweight, prolonged working hours, and shift work were statistically significantly lower among people in the group associated with heavy lifting than they were in the comparison group. These differences are likely to bias our estimate slightly in the opposite direction of the hypothesis, which decreases the probability of a false positive finding.

Another aspect of the study that needs to be considered is the possibility of detection or referral bias. The perceived adverse effects of a heart condition might increase with heavy lifting activities. Hence, the probability of becoming aware of and the propensity to seek medical treatment for a minor IHD might be higher among people who are exposed to heavy lifting at work than it is among other blue-collar workers. This potential source of bias points in the same direction as our hypothesis and would thereby increase the probability of a false positive finding. The probability that referral bias causes us to erroneously conclude that the data support the first of our underlying hypotheses is, however, decreased by our testing criteria, which tell us that we only are allowed to draw such a conclusion if also the rate ratio of death due to IHD is greater than one.

## Abbreviations

BMI: body mass index
DISCO-88: Denmark International Standard Classification of Occupations
DWECS: Danish work environment cohort survey
ICD-10: International Classification of Diseases, 10th revision
IHD: ischemic heart disease
NRCWE: National Research Centre for the Working Environment
RR: rate ratio
